# Partial nephrectomy: three dimensional (3D) models from preoperative computed tomography is the future to identify the exact loction of the tumor

**DOI:** 10.1590/S1677-5538.IBJU.2018.05.01

**Published:** 2018

**Affiliations:** 1 Professor Titular, Unidade Urogenital da Univ. Est. do Rio de Janeiro - UERJ, RJ, Brasil; 2 Urologista do Hospital da Lagoa Federal, Rio de Janeiro, RJ, Brasil; 3 Editor Associado do International Braz J Urol

The September-October 2018 issue of the International Braz J Urol presents original contributions with a lot of interesting papers in different fields: Renal stones, Prostate Cancer, Renal Cell Carcinoma, Laparoscopy, Vaginal Prolapse, BPH, Varicocele, Testicular Torsion, Renal Cystis, Basic Research, HPV, Bladder Cancer, Bladder augmentation and Overactive bladder. The papers come from many different countries such as Brazil, USA, China, Turkey, Korea, Spain, Mexico, Iran and Israel, and as usual the editor s comment highlights some papers. We decided to comment the paper about a very interesting topic: Clinical applications of personalized 3D kidney model.

Doctor Lee and collegues from Korea performed on page 950 an interesting study about three dimensional (3D) Kidney model applied to partial nephrectomy. The authors evaluated the clinical usefulness of 3D-printed renal model in performing partial nephrectomy (PN) and also in the education of medical students. They prospectively produced personalized renal models using 3D-printing methods from preoperative computed tomography (CT) images in a total of 10 patients. Two different groups (urologist and student group) appraised the clinical usefulness of 3D-renal models by answering questionnaires. After application of 3D renal models, the urologist group gave highly positive responses in asking clinical usefulness of 3D-model among PN (understanding personal anatomy: 8.9 / 10, preoperative surgical planning: 8.2 / 10, intraoperative tumor localization: 8.4 / 10, plan for further utilization in future: 8.3 / 10, clinical usefulness in complete endophytic mass: 9.5 / 10. The student group located each renal tumor correctly in 47.3% when they solely interpreted the CT images. After the introduction of 3D-models, the rate of correct answers was significantly elevated to 70.0% (p <0.001). The subjective difficulty level in localizing renal tumor was also significantly low (52% versus 27%, p < 0.001) when they utilized 3D-models. The authors concluded that the personalized 3D renal model was revealed to significantly enhance the understanding of correct renal anatomy in patients with renal tumors in both urologist and student groups. These models can be useful for establishing the perioperative planning and also education program for medical students.

The 3D printed technology is a new technology and had a lot of applications in kidney surgery (flexible ureteroscopy, endourologic procedures and partial nephrectomy). To perform a partial nephrectomy, via conventional route, laparoscopically or robotic, the most relevant issue is the understanding of intra-renal anatomy (1). In the present paper the authors shows the importance of 3D technology to understand the renal anatomy before the surgery.

Perfect knowledge and identification of intra-renal arterial anatomy may allow complete removal of the zone affected by the tumor, with maximal preservation of functioning renal parenchyma. The understanding of intravascular renal anatomy is one of the most important factors for performing the zero-ischemia robotic and laparoscopic partial nephrectomy (2).

A technique of anatomic vascular microdissection of renal artery branches is performed to selectively devascularize only the tumor, maintaining normal perfusion of the remaining kidney (3, 4). The anatomy of the renal artery is very important to understand and proceed these surgical tecnhiques. The main renal artery divides into segmental arteries near the renal hilum in five branches: Posterior; apical, upper, middle, and lower anterior segmental arteries. The apical and lower anterior segmental arteries supply the anterior and posterior surfaces of the upper and lower renal poles, respectively; the upper and middle segmental arteries supply the remainder of the anterior surface. The segmental arteries then course through the renal sinus and branch into the lobar arteries. Further divisions include the interlobar, arcuate, and interlobular arteries (5, 6).

The importance of proportional analyses of the kidney arterial segments (7) and the upper and lower pole nephrectomy in experimental models using human kidneys was studied with an accurate analysis of the vascular damage applied to partial nephrectomy (8, 9). Information about local anatomy and the probable place where injuries are more significant are crucial to the surgeon. The three dimensional models from preoperative computed tomography is the future to identify the exact location of the tumor and to perform the best surgical strategy to remove the tumor with renal parenchymal preservation.
